# Present and Future Climatic Suitability of *Castilleja tenuiflora* in Mexico

**DOI:** 10.3390/plants15192935

**Published:** 2026-09-25

**Authors:** Luis Rafael Garibay-Castro, Alma Rosa López-Laredo, Elizabeth Rubio-Rodríguez, Gabriela Trejo-Tapia, José Luis Trejo-Espino

**Affiliations:** 1 Centro de Desarrollo de Productos Bióticos, Instituto Politécnico Nacional (CEPROBI-IPN), Carr. Yautepec-Jojutla Km 6, Calle CEPROBI No. 8, Col. San Isidro, Yautepec 62739, Mexico; lr.garibayc@gmail.com (L.R.G.-C.); arlopez@ipn.mx (A.R.L.-L.); lizrubio6@gmail.com (E.R.-R.); gttapia@ipn.mx (G.T.-T.); 2 Secretaría de Ciencia, Humanidades, Tecnología e Innovación (SECIHTI), Mexico City 03940, Mexico; 3 Red de Biotecnología, Red de Medio Ambiente, Instituto Politécnico Nacional, Mexico City 07738, Mexico

**Keywords:** MaxEnt, climate change scenarios, montane medicinal plant, root hemiparasites, Mexican Protected Natural Areas (MPNAs)

## Abstract

Climate change can reshape the climatic conditions available to mountain plants. Using MaxEnt, we modelled the current and future climatic suitability of *Castilleja tenuiflora* Benth., a medicinal hemiparasitic plant, across Mexico and quantified its spatial overlap with Mexican Protected Natural Areas (PNAs). The final model (LQ, RM = 0.5), calibrated with 194 spatially filtered presence records and seven bioclimatic predictors, achieved a mean spatial-validation AUC of 0.794 ± 0.093. Annual mean temperature and temperature seasonality were the dominant predictors, jointly accounting for approximately 96% of model contribution. Current climatic suitability above the 10th-percentile training-presence threshold (P10 = 0.162865) covered approximately 548,819 km^2^. Future projections using CMCC-ESM2 under SSP1-2.6 and SSP5-8.5 for three periods spanning 2021–2080 showed net contractions in climatically suitable area under all scenarios. By 2061–2080, the area above P10 decreased by 41.6% under SSP1-2.6 and by 65.3% under SSP5-8.5. Across all projections, losses of climatically suitable area substantially exceeded gains, while the area maintaining suitable conditions decreased towards the end of the century; proportionally greater contractions were observed at more restrictive suitability thresholds. Currently, approximately 48,690 km^2^ of ≥P10 climatic suitability overlaps the PNA network, decreasing to 23,638 km^2^ under SSP5-8.5 by 2061–2080. Nevado de Toluca, Iztaccíhuatl-Popocatépetl, and Pico de Orizaba consistently retained areas above the P90 suitability threshold under current conditions and all six future projections. These results indicate a substantial potential contraction of climatic suitability for *C. tenuiflora* and highlight protected areas retaining suitable climatic conditions as priorities for field verification, monitoring, and conservation planning.

## 1. Introduction

Climate change affects biodiversity through variations in temperature and precipitation, along with increased atmospheric CO_2_ concentrations, which affect plants’ morphological, physiological, and developmental processes [1,2]. As a result, the distribution limits of many plant species may reorganize geographically, with ranges contracting, expanding, or shifting toward higher latitudes or elevations [3,4,5]. This realignment leads to increased interspecific competition [6,7], as well as local population contractions and extinctions among populations located at the warmest limits of their ranges [3,6]. In fact, plants adapted to temperate or cold climates are expected to experience significant contractions in their geographic ranges [5,6].

To assess how climate change alters a region’s environmental conditions—and potentially species distributions—ecological niche modelling has been employed. This approach uses geographic occurrence data (species presence) and regional climatic variables (past, present, or projected) to predict areas with specific environmental suitability [7,8,9]. The maximum entropy (MaxEnt) model is particularly suitable when occurrence data consist primarily of presence records, as it relates these records to environmental conditions available within a defined background area to estimate relative habitat suitability [8,9]. This approach has been used to identify suitable areas and support conservation assessments for plant species with restricted geographical distributions [10]. However, MaxEnt estimates statistical associations between species occurrences and environmental predictors. Its outputs should therefore be interpreted as estimates of relative environmental or climatic suitability, rather than as evidence of direct ecological mechanisms or as representations of the realized distribution of a species [9]. The environmental suitability of a species determines a potential area for its current or future distribution; however, its ecological suitability requires the consideration of biotic factors, such as hosts (in the case of hemiparasitic plants), pollinators, and seed dispersers, as well as specific biological characteristics [9,11]. Future climatic conditions can be explored within the Shared Socioeconomic Pathways (SSPs) of CMIP6, which combine socioeconomic development trajectories with different levels of radiative forcing [12,13]. These scenarios span contrasting futures, from lower-emission pathways such as SSP1-2.6 to fossil-fuel-intensive development under SSP5-8.5, associated with increasingly greater levels of global warming by the end of the century [14]. This framework allows exploration of potential changes in species’ climatic suitability under alternative future conditions. However, models based exclusively on bioclimatic predictors do not explicitly account for local land-use change, habitat degradation, human-footprint intensity, or other ecological factors that may constrain a species’ realized distribution.

Mexico exhibits significant topographic, climatic, and biogeographic heterogeneity, enabling it to support exceptionally high species richness. One conservation strategy adopted by the country is the establishment of 232 Protected Natural Areas (PNAs); these constitute a key spatial instrument (covering 98,000,719 hectares) for protecting biodiversity, maintaining ecosystem services, and mitigating pressures arising from habitat transformation [15]. However, the fixed boundaries of PNAs contrast with the dynamic nature of species’ responses to climate change, as climatically suitable areas may contract, shift to higher elevations, or shift beyond protected-area boundaries. Consequently, a species’ current presence within a PNA does not guarantee the continued availability of suitable climatic conditions there in the future [16,17]. This issue is particularly critical for species associated with mountainous environments, given reduced available space and the potential isolation of suitable areas as elevation increases.

Nevertheless, Mexican Protected Natural Areas (MPNAs) can preserve climatically stable refugia, alleviate certain non-climatic pressures, and help maintain landscape-level ecological connectivity [16,17]. Some studies have demonstrated that integrating ecological niche models, future climate projections, and PNA boundaries makes it possible to estimate the current and future representation of suitable areas and to identify protection gaps [18,19]. While such analyses do not constitute a comprehensive assessment of PNA management effectiveness, they do allow estimation of the network’s potential capacity to conserve suitable climatic conditions. Therefore, analyzing the overlap between species’ climatic suitability and the MPNA network will help identify potential climate refugia, stable protected areas, and unprotected suitable zones, thereby guiding conservation priorities under climate change scenarios.

*Castilleja tenuiflora* Benth. belongs to the family Orobanchaceae; it is a perennial root-hemiparasitic plant widely distributed across Mexico, the southern United States, and northern Guatemala [20], although reports of sightings in the latter two regions have declined considerably over the past 20 years [21]. It is associated with pine–oak forests, grasslands, and xerophilous scrublands, thriving under temperate and semi-arid climatic conditions across a broad elevational gradient (740–3400 m a.s.l.) [20,22]. Plants can reach up to 1 m in height and feature pubescent stems, narrow leaves, and flowers clustered in terminal inflorescences ranging in colour from yellow to orange; the fruits are capsules containing very small, reticulated seeds [20,22]. It is important in traditional Mexican medicine, with records of its use dating back to the 16th century for ailments such as coughs, stomach aches, menstrual pain, and “nerves” [23,24,25]. Regarding its phytochemistry, the plant synthesizes specialized metabolites from various chemical groups, including glycosylated iridoids, glycosylated phenylethanoids, lignans, and flavonoids [26,27]; pharmacological studies have also shown that plant extracts exhibit anti-ulcerogenic, anti-inflammatory, and cytotoxic activities [23,24,25].

As a facultative root hemiparasite, it can photosynthesize and obtain and exchange water, nutrients, organic compounds, and even genetic material through its haustorial connection to host roots [28]. This characteristic is important because it defines the ecological significance of hosts in relation to the presence of *C. tenuiflora* and indicates that climatic suitability does not guarantee the plant’s distribution and survival. Despite the available ecological, ethnobotanical, and pharmacological information, the potential future effects of climate change on conservation of *C. tenuiflora* remain insufficiently assessed. It is unknown whether climatically suitable areas will shrink, shift to higher elevations, or become progressively fragmented, and whether the current MPNAs will maintain suitable climatic conditions for the species. Based on the foregoing, this study aimed to estimate the current and future climatic suitability of *C. tenuiflora* in relation to its distribution in Mexico and to evaluate its representation within the MPNA network. We developed models using 193 occurrence records, selected bioclimatic predictors, and the MaxEnt algorithm. We projected future climatic suitability under SSP1-2.6 and SSP5-8.5 pathways for three periods: 2021–2040, 2041–2060, and 2061–2080. Specifically, the following research questions were addressed: (1) What is the current potential distribution of *C. tenuiflora*, and which bioclimatic variables are most closely associated with climatic suitability? (2) How are the extent, elevational distribution, and spatial configuration of suitable areas projected to change under contrasting climate pathways and different future periods? (3) To what extent are current, future, and climatically stable suitable areas represented within MPNAs?

## 2. Materials and Methods

### 2.1. Modelling the Climatic Suitability of C. tenuiflora in Mexico

#### 2.1.1. Study Area and Projection Extent

The study covered mainland Mexico, extending approximately from longitudes −118.365120 to −86.710403 and latitudes 14.532098 to 32.718655. Mexico’s complex topography results from its tectonic and geological history, featuring major mountain systems such as the Sierra Madre Occidental, Sierra Madre Oriental, Sierra Madre del Sur, and the Trans-Mexican Volcanic Belt [29]. These systems create steep elevational gradients and significantly influence the spatial distribution of climatic conditions across the country.

Mexico exhibits considerable climatic heterogeneity, ranging from predominantly dry conditions in the north to humid and sub-humid climates in the south and southeast. Dry and sub-humid climates prevail along the Pacific slope, whereas more humid conditions occur in parts of the Gulf of Mexico slope. Tropical climates dominate coastal zones and low-lying depressions, while temperate and cold climates are found primarily in mountainous regions, including areas exceeding 4000 m a.s.l. [29]. This climatic diversity is determined by the country’s geographic configuration, complex topography, atmospheric circulation, and the seasonal shift in the subtropical high-pressure belt [30,31,32].

From a biogeographic perspective, Mexico is situated between the Nearctic and Neotropical regions. The Nearctic component predominates in northern Mexico and many highland and mountainous areas, while Neotropical affinities are more pronounced in the south of the country and on the Yucatán Peninsula. This transitional position has contributed to the exchange and diversification of the biota of both regions [33].

The geographic extent of Mexico was defined to map current and future climatic suitability. The calibration area used for model fitting and background point selection was more restricted and was defined independently as an operational approximation of the accessible area (M), as described below.

#### 2.1.2. Occurrence Records and Spatial Filtering

An initial dataset of 371 georeferenced occurrence records for *C. tenuiflora* in Mexico was compiled from iNaturalist, the National Herbarium of Mexico (MEXU) [34], the Herbarium of the Autonomous University of the State of Morelos (UAEM), Mexican Herbaria Network [35], and field observations. For iNaturalist, only observations classified as “research grade” [36] were retained. Records were examined sequentially to remove duplicates, occurrences outside the environmental coverage, records lacking bioclimatic data, and multiple occurrences within the same 30-arc-second grid cell (“raster”), resulting in 278 validated occurrence records (Figure S1).

To further reduce spatial clustering, independent filtering procedures were applied at 5 km and 10 km, using distances calculated on the WGS84 ellipsoid via Vincenty’s inverse method [37]. Records separated by a distance less than the corresponding filter threshold were considered spatial conflicts. Record selection was based on an a priori hierarchy: MEXU > UAEM > Northwest Mexico Herbarium Network > field observations > iNaturalist; ties were resolved by prioritizing records with fewer spatial conflicts and, when necessary, based on their original order in the audited dataset. The 5 km filter retained 194 records and was used in the main analyses. In contrast, the more restrictive 10 km filter retained 161 records and was employed in a sensitivity analysis regarding the spatial filtering distance (Table S1).

#### 2.1.3. Calibration Area and Background Selection

The calibration area (M) was defined as the union of the seven biogeographic provinces containing at least one of the 278 validated records: Chihuahuan Desert, Sierra Madre Occidental, Sierra Madre Oriental, Neovolcanic Axis (Trans-Mexican Volcanic Belt), Sierra Madre del Sur, Pacific Lowlands, and Balsas Basin, following Morrone et al. (2017) [38]. This operational calibration area covered approximately 1,247,378.87 km^2^. Current environmental rasters were cropped and masked, and subsequently, 10,000 background locations were randomly selected from cells with complete environmental data, using a fixed seed of 4475314. The same background sample was used for both the 5 km and 10 km presence datasets.

#### 2.1.4. Bioclimatic Predictors and Multicollinearity Assessment

Initially, nineteen bioclimatic variables (Bio1–Bio19) from WorldClim v2.0 (1970–2000) were considered as candidate predictors, with a resolution of 30 arc-seconds [39]. A Pearson correlation analysis was performed using raster cells with complete environmental data within the calibration area, and highly correlated predictor pairs were examined (Figure 1). Decisions on which variables to retain considered correlation structure, ecological relevance, interpretability, redundancy, and transferability to future climatic conditions; Table S2 documents individual retention and exclusion decisions.

This selection process resulted in seven predictors: mean annual temperature (Bio1), mean diurnal range (Bio2), temperature seasonality (Bio4), precipitation of the driest month (Bio14), precipitation seasonality (Bio15), precipitation of the wettest quarter (Bio16), and precipitation of the coldest quarter (Bio19). Residual multicollinearity was assessed using variance inflation factors (VIF), with a VIF < 5 considered acceptable. All retained predictors fell below this threshold (1.456–4.885), and no additional variables were removed (Table S2).

#### 2.1.5. MaxEnt Calibration, Model Selection and Evaluation

Models were calibrated using MaxEnt 3.4.4 [40] and ENMeval 2.0.5.2 [41,42] in R 4.5.3 [43]. Five feature classes—linear (L), linear–quadratic (LQ), hinge (H), linear–quadratic-hinge (LQH), and linear–quadratic-hinge-product (LQHP)—were combined with eight regularization multipliers (RM; ranging from 0.5 to 4.0 in 0.5 increments), resulting in 40 candidate parameterizations. The entire fitting procedure was applied independently to datasets spatially filtered at 5 km (194 records) and 10 km (161 records), using the same set of predictors and the same 10,000 background locations within area M. Models were run for a maximum of 5000 iterations with the “clamping” option enabled and without removing additional duplicates, given that record filtering had already been performed.

Model evaluation was conducted using four spatial partitions “folds” generated via ENMeval’s latitudinal–longitudinal block approach [41,42]; each partition was evaluated using models calibrated with the remaining three partitions. Candidate parameterizations were ranked primarily based on the corrected Akaike Information Criterion (AICc), using AICc weights, spatial validation AUC, the Continuous Boyce Index (CBI), the difference between training and validation AUC, fold-specific 10th-percentile omission rates, and model complexity as complementary diagnostic metrics. The partition-specific 10th-percentile thresholds used to assess omission differed from the P10 threshold subsequently derived from the final model and used to classify current and future climatic suitability. Table S3 presents the full model-fitting results for both spatial filtering distances.

The LQ configuration with RM = 0.5 was independently selected for both datasets. The 5 km dataset was retained for the main analyses to preserve a larger sample of occurrences after spatial filtering, while the 10 km dataset was used for a more restrictive spatial thinning sensitivity analysis; the performance of the two selected models is summarized in Table S4. Fold-specific training and validation AUC values, together with their mean and standard deviation, are provided in Table S5. The final model was fitted using the 194 occurrences retained after 5 km filtering and the full set of 10,000 background locations within M. Predictions were generated from the complementary log-log (cloglog) output and interpreted as relative climatic suitability.

#### 2.1.6. Current Climatic Suitability and Threshold Definition

The selected model was projected onto the current bioclimatic layers covering the entire land area of Mexico using the complementary log–log (cloglog) output, interpreted as relative climatic suitability. Suitability values were extracted from 194 training occurrences based on the current projection using the terra package in R [44]. The 10th percentile threshold of training present was calculated using stats::quantile() with type = 7, utilizing the same set of suitability values from occurrence points to derive additional percentile thresholds (P20–P90). Subsequently, the 10th percentile threshold of training presence (P10 = 0.162865) was adopted as the primary operational threshold, including occurrence with values equal to the threshold. The additional thresholds were P20 = 0.262694, P30 = 0.389265, P40 = 0.624040, P50 = 0.749705, P60 = 0.834988, P70 = 0.914862, P80 = 0.955831, and P90 = 0.991989.

The current suitability raster was classified into ten percentile-based categories: <P10, P10–P20, P20–P30, P30–P40, P40–P50, P50–P60, P60–P70, P70–P80, P80–P90, and ≥P90. P10 was used as the primary threshold for analyses of change between the present and the future. At the same time, P50, P70, and P90 served as progressively more restrictive thresholds for sensitivity analyses and complementary analyses regarding environmental, elevational, and protected area variables. All thresholds were derived from the final current model and applied without modification to the future projections.

#### 2.1.7. Environmental Characterization of Current Climatic Suitability

The current environmental context of climate suitability was characterized using thematic layers from the CONABIO Geoportal [45]. Potential vegetation was represented by Rzedowski’s Potential Vegetation of Mexico layer [46], while soil moisture regimes were represented by the Maples-Vermeersch soil moisture regimes layer [47]. These layers provided descriptive cartographic context only; areas of suitability ≥P10 and ≥P90 were overlaid to contrast the broadest and most restrictive portions of the current climate suitability pattern.

The climatic context was assessed using the Köppen–García climate classification modified for Mexico [48]. Climate categories were spatially compared against suitability thresholds of ≥P10, ≥P50, ≥P70, and ≥P90, and the overlapping area for each climate type was expressed in km^2^ and as a percentage of the corresponding national suitability area. Table S6 presents the complete results for all overlaid climate types. In contrast, the main-text summary includes the five climate types with the greatest overlap at the ≥P10 and ≥P90 levels, listing duplicate categories only once.

#### 2.1.8. Future Climatic Projections and Changes in Climatic Suitability

Future climate suitability was projected using WorldClim v2.1 CMIP6 bioclimatic data at 30-arc-second resolution, based on the CMCC-ESM2 general circulation model and the SSP1-2.6 and SSP5-8.5 scenarios for 2021–2040, 2041–2060, and 2061–2080 [49]. For all projections, the same seven predictor variables used in the model calibration were maintained. Future layers were harmonized with current calibration rasters regarding grid geometry and numerical scale by multiplying Bio1, Bio2, and Bio4 by 10, whereas Bio14, Bio15, Bio16, and Bio19 did not require rescaling. The selected MaxEnt model (LQ, RM = 0.5) was applied without modification to the six future climate combinations—without recalibration—and predictions were generated on the “cloglog” scale to represent relative climatic suitability.

Quantification of changes between the present and the future was performed using the P10 threshold from the current model (0.162865), applied unchanged to all future projections. Raster cells were classified as unsuitable in both periods, using the 10th percentile (P10) as a reference. Areas of persistence, loss, and gain were calculated in km^2^, and net change was expressed both as the area gained minus the area lost and as a percentage of the corresponding current suitable area. Comparisons were restricted to the common valid raster domain to avoid artefacts caused by differences in “NoData” coverage. A sensitivity analysis was also conducted using thresholds of ≥P50, ≥P70, and ≥P90, applying the same thresholds derived from current conditions (Table S7).

Additionally, climatic novelty was assessed for each future projection by comparing the seven predictor values of each future cell with their corresponding ranges under current conditions within the calibration area M. Cells with at least one predictor outside its calibration range were classified as climatically novel [50]. We summarized their extent as a percentage of the analyzed future domain. This analysis was conducted independently of the suitability threshold change analysis, and the MaxEnt projections were not modified.

#### 2.1.9. Elevational Distribution of Climatic Suitability

The elevational analysis of climatic suitability was conducted in R using the terra package [44] and a digital elevation model (DEM) derived from the Mexican Elevation Continuum 3.0 (CEM 3.0) provided by the National Institute of Statistics and Geography (INEGI) [51]. The DEM used for the analysis had an approximate spatial resolution of 90 m; mean elevation was calculated and aligned with the ~30-arc-second climatic suitability grid. For current conditions and each future projection, we calculated the area-weighted mean elevation in cells meeting the suitability thresholds of ≥P10, ≥P50, ≥P70, and ≥P90. Elevational change was calculated as the difference between each future mean elevation and the corresponding current mean at the same threshold.

#### 2.1.10. Climatic Suitability in Protected Natural Areas

We evaluated current and future climatic suitability within the Mexican federal network of Protected Natural Areas (ANP) using the official CONANP layer [52]. After verifying the geometry, 182 valid ANP units were retained. We quantified suitability at the ≥P10, ≥P50, ≥P70, and ≥P90 thresholds under current conditions and across the six future projections.

Raster–polygon overlap was calculated directly in R using terra [44], sf [53], and exactextractr [54] packages. Cell areas were obtained in km^2^ using terra::cellSize(), and partial intersections along PNA boundaries were accounted for using fractional cell coverage from exactextractr::exact_extract(). PNAs were also combined into a single network geometry to estimate unique suitable area without double counting overlaps, while individual geometries were retained for PNA-specific estimates. For each threshold, national representation was calculated as the proportion of the total climatically suitable area in Mexico occurring within the PNA network.

PNAs exhibiting a non-zero climatically suitable area above a given threshold—under both current conditions and the six future projections—were classified as consistently suitable. Table S8 presents detailed results by PNA, threshold, SSP, and period.

## 3. Results

### 3.1. Current Climatic Suitability of C. tenuiflora

Under current climatic conditions, areas above the 10th-percentile training-presence threshold (P10 = 0.162865) covered approximately 548,818.89 km^2^ in Mexico (Figure 2). Climatic suitability showed a broad but spatially heterogeneous distribution, with progressively higher suitability classes becoming increasingly restricted. Areas above P50 covered 76,364.35 km^2^, whereas 29,793.61 km^2^ remained above P70 and 5387.84 km^2^ above P90. The highest suitability classes were concentrated predominantly in the mountainous regions of central Mexico. In contrast, lower and intermediate percentile classes extended more broadly across the country and formed discontinuous areas outside this central concentration. Areas ≥P90 were comparatively restricted and occurred mainly within the central portion of the current suitability pattern, with smaller discontinuous patches elsewhere.

The final MaxEnt model (LQ feature classes; regularization multiplier = 0.5) achieved a mean spatial-validation AUC of 0.794 ± 0.093 across the four spatial validation folds (Figure 3a). Individual validation AUC values were 0.884, 0.825, 0.664, and 0.805 for Blocks 1–4, respectively. Annual mean temperature (Bio1) and temperature seasonality (Bio4) were the dominant contributors, accounting for 50.30% and 45.71% of the total percent contribution, respectively, and jointly representing approximately 96.0% of the model contribution (Figure 3b). The remaining variables contributed substantially less: mean diurnal range (Bio2, 2.24%), precipitation of the wettest quarter (Bio16, 1.36%), precipitation of the driest month (Bio14, 0.25%), precipitation seasonality (Bio15, 0.09%), and precipitation of the coldest quarter (Bio19, 0.06%). The response curves of the two dominant variables showed clear changes in relative climatic suitability along their respective gradients (Figure 3c,d). For annual mean temperature (Bio1; Figure 3c), relative climatic suitability was highest at the lower end of the evaluated temperature range, reaching approximately 0.64 at 5.6 °C. Suitability remained above 0.60 up to approximately 8 °C and declined progressively thereafter, reaching about 0.56 at 10 °C, 0.42 at 14 °C, 0.25 at 18 °C, and 0.10 at approximately 22 °C. At temperatures above 25 °C, relative climatic suitability was very low, approaching zero towards the upper end of the evaluated range. For temperature seasonality (Bio4; Figure 3d), the highest relative climatic suitability values occurred at the lowest seasonality values evaluated, reaching approximately 0.79 at 38–46 SD × 100. Suitability decreased markedly as seasonality increased, falling to approximately 0.65 at 100, 0.50 at 150, 0.37 at 200, and 0.20 at approximately 300 SD × 100. Relative climatic suitability reached its lowest values, approximately 0.08, around 600 SD × 100, followed by a slight increase towards the upper end of the evaluated range, reaching approximately 0.10 near 780 SD × 100.

We overlaid current climatic suitability for *C. tenuiflora* with the main vegetation types in Mexico to characterize the environmental context of the modelled suitable areas (Figure 4). Areas above the P10 threshold overlapped several vegetation types, including conifer and oak forests, grasslands, xerophilous scrub, thorn forests, and tropical deciduous forests. This broad spatial overlap reflects the extensive geographical distribution of ≥P10 climatic suitability. In contrast, areas above the more restrictive P90 threshold were considerably more spatially concentrated, occurring mainly in the mountainous regions of central Mexico, where they overlapped predominantly with conifer and oak forests and, locally, with other vegetation types.

Conifer and oak forests in Mexico comprise vegetation dominated principally by species of *Pinus* and *Quercus*, with *Abies*-dominated forests occurring predominantly under colder and more humid conditions at higher elevations [22]. Climatically suitable areas also showed local spatial correspondence with cloud forests, which are generally associated with humid mountain environments, slopes, ravines, and valleys characterized by frequent cloud cover and high atmospheric moisture [22]. At the broader ≥P10 threshold, suitability additionally extended into warmer and drier vegetation formations, including tropical deciduous forests and xerophilous scrub. These spatial correspondences are presented as environmental context for the climatic suitability model rather than as evidence of vegetation dependence or as an independent validation of the model.

We also examined the current climatic suitability of *C. tenuiflora* in relation to soil moisture regimes across Mexico (Figure 5). Areas above the P10 threshold occurred across a broad range of moisture conditions, extending from xeric and ustic regimes to udic conditions and, locally, into aridic areas. This pattern reflects the broad geographical extent of the ≥P10 suitability threshold, which spans environments with contrasting annual periods of soil moisture availability.

In contrast, areas above the more restrictive P90 threshold were much more spatially concentrated, particularly in central Mexico. Many of these areas overlapped with ustic and udic soil moisture regimes, corresponding approximately to 180–330 or more days per year with available soil moisture. Relatively few ≥P90 areas were evident within the drier aridic and xeric regimes of northern and north–central Mexico. As with the vegetation overlay, these spatial associations are presented as environmental context for the climatic suitability model rather than as an independent validation of the model.

The current climatic suitability of *C. tenuiflora* overlapped a broad range of climate types across Mexico, although their relative contribution differed markedly between the ≥P10 and ≥P90 thresholds (Table 1). At the ≥P10 threshold, the greatest spatial overlap occurred with the temperate semi-arid climate BS1kw, which accounted for 93,162.67 km^2^ (16.98% of the national ≥P10 area). This was followed by the temperate subhumid climate C(w1) with 53,101.11 km^2^ (9.68%), the semi-warm subhumid climate (A)C(wo) with 49,092.23 km^2^ (8.95%), the temperate subhumid climate C(w2) with 48,191.07 km^2^ (8.78%), and the semi-warm subhumid climate (A)C(w1) with 43,488.74 km^2^ (7.92%). Together, these five climate types represented approximately 52.3% of the total ≥P10 climatically suitable area. Nevertheless, ≥P10 suitability extended across a much broader climatic spectrum, overlapping 55 climate categories in the complete analysis (Table S6).

The dominant ≥P10 climate types encompassed contrasting thermal and moisture conditions. BS1kw corresponds to a temperate semi-arid climate with summer rainfall, whereas C(w1) and C(w2) are temperate subhumid climates with annual mean temperatures of approximately 12–18 °C and summer-dominated rainfall. Within this temperate subhumid group, C(w2) represents wetter conditions than C(w1), whereas (A)C(wo) and (A)C(w1) correspond to semi-warm subhumid climates with annual mean temperatures above 18 °C and differing levels of effective moisture. Table S6 provides detailed temperature and precipitation criteria for all climate categories.

In contrast, the ≥P90 suitability threshold showed a much stronger climatic concentration. The semi-cold subhumid climate Cb′(w2), characterized by a long cool summer and annual mean temperatures of approximately 5–12 °C, contained 3575.31 km^2^ of ≥P90 suitability, equivalent to 66.36% of the national ≥P90 area. The next largest overlaps occurred with C(w2) (853.61 km^2^; 15.84%) and C(w1) (534.78 km^2^; 9.93%). Together, these three climate types accounted for approximately 92.1% of the total ≥P90 area. The remaining ≥P90 suitability was distributed among several humid, subhumid, semi-cold, and cold climate categories, each contributing comparatively small areas.

### 3.2. Projected Changes in Climatic Suitability Under Climate-Change Scenarios

Future projections showed a net contraction of climatically suitable area for *C. tenuiflora* under all six combinations of SSP and time period evaluated (Table 2). Using the 10th-percentile training-presence threshold (P10 = 0.162865) and the current ≥P10 area within the common present–future raster domain (548,817.42 km^2^) as the reference, projected suitable area under SSP1-2.6 decreased to 410,665.71 km^2^ in 2021–2040, 335,352.41 km^2^ in 2041–2060, and 320,593.60 km^2^ in 2061–2080. These changes corresponded to net reductions of 25.17%, 38.90%, and 41.58%, respectively. Under SSP5-8.5, ≥P10 suitability covered 430,744.06 km^2^ in 2021–2040, 289,165.89 km^2^ in 2041–2060, and 190,692.25 km^2^ in 2061–2080, representing net reductions of 21.51%, 47.31%, and 65.25%, respectively.

In all projections, losses of currently suitable area exceeded gains (Table 2). Under SSP1-2.6, projected losses increased from 151,792.68 km^2^ in 2021–2040 to 236,052.86 km^2^ in 2061–2080, whereas gains decreased from 13,640.97 to 7829.04 km^2^. Consequently, persistent ≥P10 suitability declined from 397,024.74 to 312,764.56 km^2^ over the same period. Under SSP5-8.5, the difference between losses and gains became progressively greater: losses increased from 138,461.33 km^2^ in 2021–2040 to 360,071.61 km^2^ in 2061–2080, while gains declined from 20,387.96 to only 1946.44 km^2^. By the final period, 188,745.81 km^2^ of the current ≥P10 suitable area remained climatically suitable under this pathway.

The spatial distribution of these changes was consistent with the quantitative pattern (Figure 6). Under SSP1-2.6, persistent suitability remained broadly distributed during 2021–2040, although losses were already evident across several portions of the current suitable range. During 2041–2060 and 2061–2080, suitability losses became more extensive, while persistent areas occupied progressively smaller portions of the current suitability pattern. Gains were limited in extent and occurred as comparatively small and discontinuous areas throughout the three periods.

Under SSP5-8.5, the spatial contrast between persistence and loss became more pronounced through time (Figure 6d–f). During 2021–2040, substantial areas of current suitability persisted, accompanied by both losses and relatively limited gains. By 2041–2060, losses occupied a larger proportion of the current suitable area and persistent suitability became more restricted. The greatest spatial change occurred in 2061–2080, when suitability loss was widespread across much of the area currently above P10, whereas persistent areas were considerably reduced and gains were scarce. This spatial pattern corresponded with the 65.25% net reduction quantified for the same scenario and period.

Sensitivity analyses using progressively more restrictive climatic-suitability thresholds showed the same general direction of change, with proportionally greater contractions at ≥P50, ≥P70, and ≥P90 than at ≥P10, particularly towards the end of the century under SSP5-8.5 (Table S7).

### 3.3. Elevational Changes in Climatic Suitability

The mean elevation in climatically suitable areas increased under all future projections relative to current conditions. However, the magnitude and temporal pattern of this change differed by suitability threshold and SSP (Table 3). Under current conditions, mean elevation increased from 2011 m a.s.l. for areas ≥P10 to 2443 m for ≥P50, 2654 m for ≥P70, and 3102 m for ≥P90, indicating that progressively more restrictive climatic suitability thresholds were associated with increasingly higher elevations.

Under SSP1-2.6, the mean elevation in ≥P10 suitability increased to 2114 m in 2021–2040, 2176 m in 2041–2060, and 2185 m in 2061–2080, corresponding to increases of 103, 164, and 173 m relative to current conditions, respectively. Greater elevational differences were observed at the more restrictive thresholds. Mean elevation at ≥P90 increased by 450 m in 2021–2040 and by 483 m in 2041–2060, reaching 3552 and 3585 m a.s.l., respectively. By 2061–2080, the mean elevation in ≥P90 suitability decreased slightly relative to the preceding periods to 3444 m, although it remained 342 m above the current value. Similar non-linear temporal patterns occurred at ≥P50 and ≥P70, which reached their highest mean elevations under SSP1-2.6 during 2041–2060 before declining slightly in the final period.

Under SSP5-8.5, mean elevation increased progressively through time at all four suitability thresholds. At ≥P10, mean elevation rose from 2115 m in 2021–2040 to 2222 m in 2041–2060 and 2342 m in 2061–2080, representing increases of 104, 210, and 331 m relative to current conditions. The upward differences became progressively larger at the more restrictive thresholds. By 2061–2080, mean elevation reached 2893 m for ≥P50, 3346 m for ≥P70, and 3931 m for ≥P90, corresponding to increases of 450, 692, and 829 m, respectively. Thus, the strongest elevational redistribution of climatic suitability occurred under SSP5-8.5 towards the end of the century, particularly for areas associated with the highest climatic suitability thresholds.

### 3.4. Representation of Climatic Suitability Within Protected Natural Areas

To evaluate the representation of climatically suitable areas for *C. tenuiflora* within the Mexican Protected Natural Area (PNA) network, the current and six future climatic-suitability projections were intersected with the PNA boundaries at the ≥P10, ≥P50, ≥P70, and ≥P90 thresholds (Table 4). Under current climatic conditions, 48,689.93 km^2^ of ≥P10 suitability occurred within PNAs, corresponding to 8.87% of the total ≥P10 climatically suitable area in Mexico. At progressively more restrictive thresholds, the area represented within PNAs decreased to 6497.76 km^2^ at ≥P50, 3786.53 km^2^ at ≥P70, and 1545.69 km^2^ at ≥P90. These areas represented 8.51%, 12.71%, and 28.69% of the corresponding national suitability areas, respectively.

Under SSP1-2.6, the ≥P10 area occurring within PNAs declined to 38,486.26 km^2^ in 2021–2040, 34,886.86 km^2^ in 2041–2060, and 34,278.91 km^2^ in 2061–2080. These values corresponded to reductions of 20.96%, 28.35%, and 29.60%, respectively, relative to the current ≥P10 area within the PNA network. The same general reduction in absolute area was observed at the more restrictive thresholds. For example, ≥P90 suitability within PNAs declined from 1545.69 km^2^ under current conditions to 379.50 km^2^ in 2021–2040, followed by 456.06 km^2^ in 2041–2060 and 763.72 km^2^ in 2061–2080. Despite these reductions in absolute area, the proportion of the remaining national ≥P90 suitability represented within PNAs ranged from 50.42% to 58.48% across the three SSP1-2.6 periods.

Under SSP5-8.5, the ≥P10 area represented within PNAs was 40,832.18 km^2^ in 2021–2040, 30,970.42 km^2^ in 2041–2060, and 23,638.25 km^2^ in 2061–2080, corresponding to reductions of 16.14%, 36.39%, and 51.45%, respectively, relative to current conditions. Reductions were also evident at the more restrictive thresholds. By 2061–2080, the area within PNAs declined to 2799.09 km^2^ at ≥P50, 850.24 km^2^ at ≥P70, and 251.75 km^2^ at ≥P90. However, because the total national climatically suitable area contracted more strongly at these restrictive thresholds, the proportion of the remaining national suitability represented within the PNA network increased to 25.89% at ≥P50, 42.97% at ≥P70, and 74.97% at ≥P90 during the same period.

The recurrence analysis further showed that the number of PNAs containing climatically suitable areas under both current conditions and all six future projections decreased as the suitability threshold became more restrictive. A total of 69 PNAs contained areas ≥P10 under all evaluated conditions, whereas 18 and 13 PNAs contained areas ≥P50 and ≥P70, respectively. Only three PNAs contained areas above the ≥P90 threshold under current conditions and in all six future projections: Nevado de Toluca, Iztaccíhuatl-Popocatépetl, and Pico de Orizaba. Detailed results for individual PNAs, thresholds, scenarios, time periods, and recurrent protected areas are provided in the Tables S8 and S9.

## 4. Discussion

### 4.1. Current Potential Distribution and Projections Under Climate-Change Scenarios

The spatially explicit evaluation used here provides a more conservative basis for interpreting the climatic suitability of *C. tenuiflora* than the random cross-validation approach initially applied to the model. The final LQ model with a regularization multiplier of 0.5 showed adequate discrimination across geographically separated validation folds, although performance varied. This variation is relevant because it indicates that model performance was not spatially uniform and cautions against interpreting a single average AUC as evidence of equivalent predictive ability throughout Mexico. Nevertheless, independently selecting the same LQ, RM = 0.5 configuration after both 5 km and 10 km spatial filtering supports the robustness of model selection to the thinning distances examined. The agreement between the analyses is particularly useful because the principal 5 km dataset retained more occurrence records.

In contrast, the more restrictive 10 km dataset provided an independent sensitivity test of spatial filtering. This approach is consistent with current recommendations to evaluate ecological niche models through explicit tuning and spatially structured validation rather than relying exclusively on discrimination metrics or default parameterizations [41]. Moreover, model complexity and predictor selection can influence transferability, reinforcing the value of favouring parsimonious configurations when models are projected to new environmental conditions [55]. Kass et al. specifically emphasize tuning, reporting of parameterization, and spatial cross-validation in ENMeval, while Low et al. show that predictor complexity and feature selection affect MaxEnt transferability.

The climatic associations identified by the final model differed markedly from those emphasized in the previous modelling approach. Annual mean temperature and temperature seasonality jointly accounted for most of the model contribution, placing thermal conditions at the centre of the fitted climatic relationship. Relative climatic suitability was greatest towards the cooler portion of the annual-mean-temperature gradient and declined progressively as temperature increased. A similar decline occurred with increasing temperature seasonality. These responses are compatible with the predominantly montane environments in which *C. tenuiflora* has been studied in central Mexico [20,56], but they should not be interpreted as direct physiological response functions. MaxEnt contributions and response curves describe associations between predictors and the occurrence–background data used to fit the model [9]; they do not establish thermal tolerance limits or demonstrate that temperature operates independently of other environmental conditions.

The much lower contribution of precipitation predictors likewise should not be interpreted as evidence that water availability is biologically unimportant for *C. tenuiflora*. Demographic work on the species has documented processes associated with seasonal environmental conditions, including germination and the persistence of individuals capable of resprouting after loss of above-ground structures [56]. These observations remain ecologically relevant, but the revised model no longer supports treating precipitation of the wettest quarter as a dominant climatic driver. Instead, precipitation, soil–water availability, and local hydrological conditions should be regarded as processes that may modify plant performance at scales not fully represented by the dominant climatic associations identified here.

The environmental overlays provide additional context for this interpretation. The broader climatic-suitability domain overlaps several vegetation formations and climatic regimes, whereas the most restrictive suitability classes are much more concentrated in the mountainous environments of central Mexico. In particular, their correspondence with temperate and semi-cold climates and with conifer and oak forests is consistent with the cool montane setting associated with the strongest climatic suitability. Mexican conifer and oak forests span a broad range of mountain environments, including *Abies*-dominated forests under colder, more humid conditions at higher elevations [22]. Local correspondence with cloud-forest environments similarly places some suitable areas in systems characterized by comparatively humid mountain conditions [22].

These relationships should nevertheless be interpreted cautiously because the vegetation layer represents potential vegetation rather than current vegetation cover, and neither vegetation nor soil-moisture regimes were predictors in the MaxEnt model. Their spatial correspondence with climatic suitability therefore provides environmental context rather than independent validation. This distinction matters for the broader ≥P10 domain, which spans vegetation types and climatic regimes from temperate forests to grasslands, xerophilous scrub, and tropical deciduous vegetation. This breadth indicates that meeting the principal climatic threshold alone cannot be assumed to imply equivalent habitat quality across the mapped area.

Local microclimatic conditions may further modify the relationship between regional climate and plant performance. Forest canopies can reduce temperature extremes relative to open environments [57], and soil moisture has been associated with temperate forest environments’ capacity to buffer maximum understorey temperatures [58]. We did not measure these mechanisms for *C. tenuiflora* in the present study, and the thematic overlays cannot demonstrate their operation. They nevertheless provide plausible hypotheses for explaining why regional climatic suitability may translate differently into local environmental conditions among forested, open or seasonally dry environments. Direct measurements of canopy structure, soil moisture, and near-ground temperature at populations of *C. tenuiflora* would therefore help test how strongly local environments modify the climatic relationships identified here.

Future projections indicate a consistent contraction of relative climatic suitability rather than the near-stability previously inferred under the lower-emission pathway. Losses exceeded gains in every SSP–period combination evaluated, and the imbalance became increasingly pronounced through time. Agreement in the direction of change across the principal P10 threshold and the progressively more restrictive P50, P70, and P90 thresholds strengthens the conclusion that contraction is not an artefact of a single operational cut-off. At the same time, the greater proportional reductions at restrictive thresholds indicate that the portion of the climatic gradient associated with the highest current suitability may be particularly sensitive to the projected changes.

The contrast between SSP1-2.6 and SSP5-8.5 becomes especially marked in the later periods. Although both pathways projected substantial contraction, losses were considerably greater by 2061–2080 under SSP5-8.5. Importantly, the ordering between pathways was not identical during the earliest period, so the results should not be interpreted as a simple monotonic relationship in which the higher-forcing pathway necessarily produces the greater change at every time step. Rather, they show increasing divergence through time within the climatic trajectories simulated by CMCC-ESM2. Responses of other montane plants similarly indicate that warming effects can vary across periods and climatic conditions rather than following a uniform trajectory; for example, *Caragana versicolor* has been projected to respond positively to moderate warming before becoming increasingly constrained under more extreme climatic conditions [59]. Such comparisons provide useful ecological context, but differences among species, regions, and modelling approaches preclude direct comparison of effect sizes.

For *C. tenuiflora*, crossing a climatic-suitability threshold represents a change in the climatic conditions associated with the fitted model, not local extinction, disappearance or future establishment. This distinction matters because the projections do not incorporate demographic persistence, dispersal, vegetation change, or biotic interactions. Likewise, areas identified as future gains indicate cells in which climatic conditions move above the selected threshold; they do not imply that populations will be able to reach or occupy those locations.

The elevational analysis adds another dimension to this redistribution. Progressively more restrictive current suitability thresholds were already associated with higher mean elevations, and future projections generally shifted climatic suitability towards still higher mean elevations. This response was strongest under SSP5-8.5 towards 2061–2080, particularly for the most restrictive suitability classes. Observed upward shifts in plant distributions and transformations of high-elevation vegetation have been documented under contemporary warming [4,5], providing relevant ecological context for this result. More recent large-scale assessments similarly indicate that elevation can strongly modulate plant responses to climate change: modelling of thousands of Cerrado angiosperms showed that montane species were particularly prone to projected range loss, whereas a synthesis for Andean vegetation emphasized both the vulnerability of high-elevation biomes and the substantial uncertainty involved in modelling plant responses in topographically complex mountain systems [60,61]. However, those studies concern realized biological responses, whereas the present analysis quantifies the elevation in modelled climatic-suitability surfaces. The projected increase in mean elevation therefore does not demonstrate migration of *C. tenuiflora*.

Nor does an upward redistribution imply a simple progressive reduction in available mountain area. The relationship between surface area and elevation varies substantially with mountain topography [62], and local establishment will additionally depend on substrates, vegetation, hosts, dispersal and demographic processes. The elevational results are therefore most appropriately interpreted as evidence that the climatic conditions represented by the model become increasingly associated with higher portions of the Mexican landscape under future scenarios.

Climatic novelty adds a further qualification to these projections. Most of the evaluated future domain remained within the individual calibration ranges of the seven predictors. Still, the proportion containing at least one value outside those ranges increased most clearly under SSP5-8.5 during the final analyzed period. These areas involve greater environmental extrapolation and consequently warrant more cautious interpretation. Transferability studies show that projecting into environmental conditions not represented during calibration entails extrapolation and can substantially alter model behaviour, making explicit assessment of environmental novelty particularly important in forecasts through space or time [63]. More generally, evidence across transferred species-distribution models indicates that changes in occupied environmental space can reduce model transferability, reinforcing the need to interpret temporal projections as conditional on the stability of the fitted climatic relationships [64]. Conversely, remaining within the individual ranges of all seven predictors does not establish complete multivariate climatic analogy, because combinations of individually familiar values can still represent climatic configurations that were uncommon or absent during calibration. Climatic novelty should therefore be regarded as an additional indicator of transfer uncertainty rather than as a binary criterion for accepting or rejecting future projections.

### 4.2. Biotic Interactions and Their Potential Relationship with the Distribution of Castilleja tenuiflora

The partial dependence of *C. tenuiflora* on other plant species introduces an ecological dimension that is not explicitly represented in the climate-based model. As a facultative root hemiparasite, the species can germinate and develop without a host, although association with other plants may favour certain components of its demographic performance [56]. Haustorial structures and five potential host species were documented in Iztaccíhuatl–Popocatépetl National Park; however, researchers did not observe direct physical contact between the haustoria and the roots of the potential hosts [20]. Consequently, an upslope shift in climatic suitability does not guarantee that the ecological conditions required for the establishment and persistence of *C. tenuiflora* will shift in the same direction or at a comparable rate. The identity, abundance, and distribution of potential hosts could modify the correspondence between projected climatic suitability and the realized distribution of the species. Nevertheless, given the generalist and facultative nature of its hemiparasitism, the absence of a particular host species should not be assumed to prevent its establishment completely. The availability of a diverse community of potential hosts may be more important than co-occurrence with any single plant species.

Interactions with floral visitors may also help explain the realized distribution and reproductive success of *C. tenuiflora*. In La Malinche National Park, six hummingbird species were recorded visiting its flowers: *Basilinna leucotis* (Vieillot, 1818), *Eugenes fulgens* (Swainson, 1827), *Archilochus colubris* (Linnaeus, 1758), *Selasphorus rufus* (Gmelin, 1788), *Selasphorus platycercus* (Swainson, 1827), and *Selasphorus sasin* (Lesson, 1829) [65]. Within that community, *C. tenuiflora* provided comparatively sparse and dispersed floral resources but was used by both resident and migratory hummingbirds. The species can produce reproductive structures throughout much of the year, with peak flowering occurring during summer and autumn [56]. They may therefore contribute to the temporal availability of floral resources in the temperate forests of central Mexico.

Studies of interaction networks support this interpretation. In La Malinche National Park, *C. tenuiflora* formed part of the core of the hummingbird–plant network, together with *Penstemon roseus* (Cerv. ex Sweet) G.Don, *Salvia elegans* Vahl, *Penstemon gentianoides* (Kunth) Poir., and *Bouvardia ternifolia* (Cav.) Schltdl. It was also identified as an important species in a network located near El Chico National Park [66]. More recently, *C. tenuiflora* was identified as a core species in the pine–oak and fir forest networks of Volcán Nevado de Colima National Park, where it provided floral resources for hummingbirds [67]. In this latter system, phenological overlap was the factor most strongly associated with interaction frequency across elevations and seasons. In contrast, species turnover and reorganized associations contributed to spatial and temporal differences among networks.

These findings suggest that temporal correspondence between *C. tenuiflora* flowering and hummingbird presence may be as important as geographical overlap. However, the available evidence does not demonstrate that all recorded visitors are effective pollinators. In *Castilleja*, floral traits associated with pollination syndromes do not always accurately predict the identity of the most frequent floral visitors [68]. Furthermore, a floral visit does not necessarily result in effective pollen deposition or increased fruit and seed production. Species recorded visiting the flowers of *C. tenuiflora* should therefore be described as floral visitors or potential pollinators until their effectiveness has been evaluated through observations of contact with reproductive structures, pollen deposition, visitor-exclusion experiments, or measurements of fruit and seed production.

Visitor composition should not be extrapolated uniformly across the entire distribution of *C. tenuiflora*. A study of 23 populations of different *Castilleja* species distributed across approximately 1900 km found that visitor assemblages included bees, butterflies, hawkmoths, and hummingbirds, and that their composition varied among species and geographical regions [69]. Although most populations exhibited relatively generalized visitation systems, the relative frequency of functional groups varied spatially. Consequently, observations from La Malinche, El Chico, or Nevado de Colima do not necessarily represent *C. tenuiflora* interactions throughout Mexico.

Generalized interactions may provide some capacity to respond to spatial or temporal variation in the abundance of a principal visitor. In *Castilleja sessiliflora* Pursh, diurnal and nocturnal exclusion experiments demonstrated that hawkmoth visitation resulted in greater fruit production in populations with long corollas. Nevertheless, secondary diurnal visitors, primarily small bees, also contributed to fruit production and provided reproductive assurance when hawkmoths were scarce [70]. Although these findings cannot be extrapolated directly to *C. tenuiflora*, they demonstrate that the local decline or absence of a particular visitor group does not necessarily result in the complete loss of pollination services. The response will depend on the degree of plant specialization, the effectiveness of alternative visitors, and the temporal correspondence between flowering and visitor activity.

At the same time, extreme climatic events can temporarily disrupt these interactions. In a temperate forest in central Mexico, an intense snowfall event reduced floral abundance from approximately 8700 to 160 flowers. It resulted in the temporary absence of both hummingbird records and floral visits. Hummingbird activity recovered rapidly during the following month, although the recovery of floral resources was slower [71]. That study did not directly evaluate the reproductive response of *C. tenuiflora*. Still, it demonstrates that extreme climatic events can simultaneously affect plants, floral visitors, and their interactions, even when species distributions do not change immediately.

Consequently, the projections in this study represent only changes in the climatic suitability of *C. tenuiflora*. They cannot predict future spatial or temporal decoupling between the plant and its floral visitors. The concentration of suitable areas at higher elevations could alter visitor assemblage composition, phenological overlap, or interaction frequency; however, these possibilities represent ecological hypotheses rather than model outputs [72,73]. Evaluating them would require separate modelling of the current and future distributions of the principal potential visitors, followed by analysis of their overlap with areas suitable for the plant. In other plant systems, incorporating the distributions of pollinators or seed dispersers into niche models has revealed areas where abiotic conditions are suitable for the plant, but where the biotic interactions required for its persistence may be limited [74,75]. This represents a relevant direction for future research on *C. tenuiflora*.

### 4.3. Representation Within Protected Areas and Ecological Implications for Conservation

The revised protected-area analysis indicates that a relatively small proportion of the broad current ≥P10 climatic-suitability domain occurs within the federal PNA network. However, representation increases at more restrictive thresholds. This pattern suggests that the existing network includes a disproportionately large share of areas associated with the highest current climatic suitability, particularly in montane central Mexico. However, interpret representation in both absolute and proportional terms. Future projections consistently reduce the absolute amount of climatically suitable area within PNAs, even when the proportion of the remaining national suitable area represented by the network increases.

This distinction matters most at restrictive thresholds under SSP5-8.5. A rising percentage of national suitability inside PNAs does not indicate that protection is becoming more effective; it can occur simply because climatic suitability outside the network contracts more rapidly than the amount remaining inside it. The protected-area results therefore describe spatial representation of modelled climatic conditions rather than management performance, habitat quality or population security. Similar concerns have been raised for protected areas elsewhere in Mexico, where climate change may alter the climatic space contained within fixed reserve boundaries [76]. More broadly, assessments of protected-area networks show that climate change can reduce future representation of plants and other species even where protected coverage is maintained or expanded [77,78]. Manrique-Ascencio et al. found climate-driven changes in plant diversity and protected-area effectiveness in Neotropical seasonally dry forests. Meanwhile, Fajardo et al. projected declining species representation in tropical-Andean protected areas under future climate scenarios.

Three PNAs—Nevado de Toluca, Iztaccíhuatl–Popocatépetl and Pico de Orizaba—contained areas above the most restrictive ≥P90 threshold under current conditions and all six future projections. This recurrence makes them particularly relevant for field verification and long-term monitoring. It does not, however, demonstrate that identical cells remain climatically suitable throughout all periods, nor that *C. tenuiflora* populations persist within those areas. Consequently, these PNAs should not be classified as demonstrated climatic refugia based on the present analysis alone. Assessing that possibility would require finer spatial analysis of climatic persistence together with observations of populations, demographic performance, habitat condition and local ecological processes [79]. This distinction is particularly pertinent in Mexico, where a recent national assessment of terrestrial animals and vascular plants showed that the relationship between protected-area coverage and extinction risk varies among taxonomic groups and concluded that the protected-area network should be evaluated alongside climate change and other environmental pressures [80].

The ecological value of these protected areas may also depend on processes extending beyond climate. In mountain systems where *C. tenuiflora* occurs, conserving local vegetation may help maintain potential host communities, microclimatic conditions, and floral-interaction networks. The documented role of *C. tenuiflora* in plant–hummingbird networks [67] further shows that conserving suitable conditions for the plant may affect interacting species. Conversely, climatic suitability within a protected boundary may provide limited conservation benefit if host communities, vegetation structure, or floral visitors change in incompatible ways. Protected-area planning for this species would therefore benefit from integrating climatic information with field-based evidence on local populations and ecological interactions.

The presence of substantial climatic suitability outside federal PNAs is equally important. Areas beyond the current network may contribute to future persistence, movement among mountain systems, or conservation under other land-management regimes, but this study did not evaluate those functions directly. Likewise, the present analysis does not include land-use change, habitat degradation, or connectivity, so the mapped areas outside PNAs should not automatically be interpreted as available habitat or dispersal corridors. Rather, they represent priorities for evaluating whether climatically suitable conditions coincide with vegetation integrity, population occurrence and feasible conservation opportunities.

The ecological significance of conserving *Castilleja* species may also extend beyond the focal plant itself, although evidence remains context-dependent. Recent reports have linked the presence of montane *Castilleja* to greater local plant diversity [81], whereas effects of hemiparasitic plants on community properties have been inconsistent across species and sites [82]. These findings do not demonstrate that *C. tenuiflora* functions as a keystone species. Still, they reinforce the value of considering hemiparasitic plants within broader community and interaction networks rather than exclusively as isolated conservation targets.

Several limitations define the scope of these conservation interpretations. Future projections were based on a single GCM, CMCC-ESM2, and consequently represent conditional outcomes rather than uncertainty across climate models. The MaxEnt model incorporated climatic predictors but not dispersal, demographic dynamics, explicit biotic interactions, land-use change, or human disturbance. Climatic novelty also indicates that part of the future domain contains conditions outside at least one calibration range, requiring additional caution in those areas. Finally, overlap with PNAs quantifies the representation of relative climatic suitability within protected boundaries but cannot establish population persistence or conservation effectiveness.

Taken together, the results provide a climatic framework for prioritizing field verification rather than forecasting future occupancy. The consistent contraction of relative climatic suitability, its redistribution towards higher mean elevations and the decline in suitable area within PNAs indicate that conservation planning for *C. tenuiflora* should not rely solely on its current climatic geography. Monitoring within recurrently suitable mountain PNAs, surveys of climatically suitable areas outside the federal network, and evaluation of host availability, demographic performance, and plant–visitor interactions would allow the climatic projections developed here to translate into more biologically informed conservation decisions.

## 5. Conclusions

Climatic suitability for *C. tenuiflora* was predominantly associated with mean annual temperature and temperature seasonality; the most restricted suitability zones were concentrated in the mountainous regions of central Mexico. Future projections indicated a steady contraction of relative climatic suitability under both the SSP1-2.6 and SSP5-8.5 scenarios, with losses exceeding gains and more pronounced proportional reductions at the most restrictive suitability thresholds. Furthermore, climatically suitable areas shifted toward higher mean elevations, particularly under the SSP5-8.5 scenario.

The climatically suitable surface area represented within the federal network of Natural Protected Areas decreased in absolute terms according to future projections, although proportional representation increased at certain restrictive thresholds as suitability contracted nationwide. The Nevado de Toluca, Iztaccíhuatl–Popocatépetl, and Pico de Orizaba zones consistently harboured areas exceeding the ≥P90 threshold—under both current conditions and all six future projections—supporting their prioritization for field verification and long-term monitoring.

Our climatic suitability study identified and defined the presence of a climate conducive to the development of *C. tenuiflora* in specific areas of Mexico, including particular natural protected areas, thereby providing important information for conservation planning. This should not be interpreted as a forecast of areas potentially suitable for its current or future distribution, but rather as valuable information to be complemented by biotic, ecological, and anthropogenic factors—such as seed dispersal processes, the presence of hosts and pollinators, demographic processes, and land-use changes, among others. It is also important to consider the uncertainty inherent in projecting future climatic conditions.

## Figures and Tables

**Figure 1:**
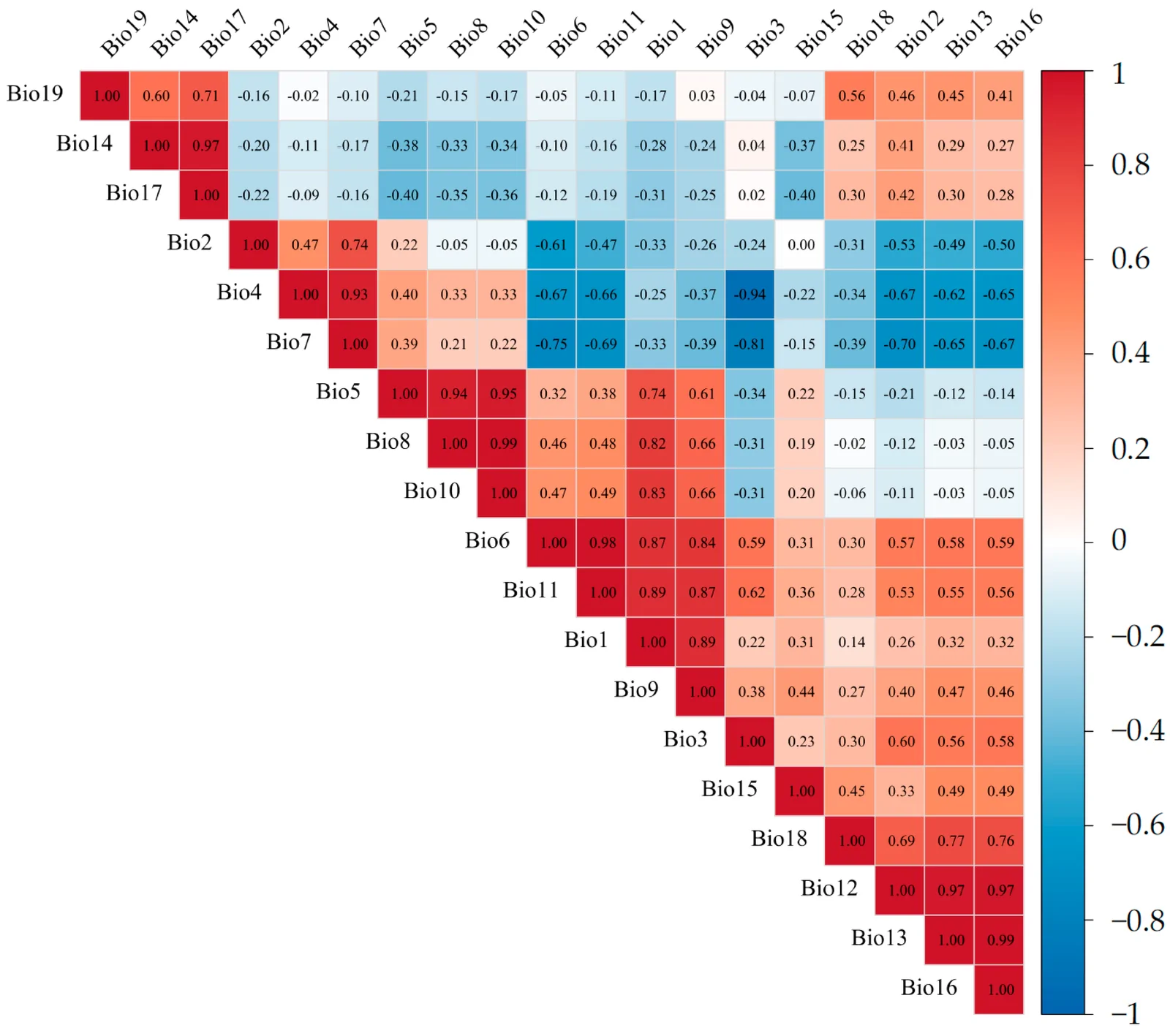
Pearson correlation matrix of the 19 candidate bioclimatic predictors within the accessible calibration area *M* of *C. tenuiflora*. Pearson correlation coefficients were calculated from raster cells with complete environmental data within *M*. Positive correlations are shown in red and negative correlations in blue, with colour intensity increasing with correlation strength. Predictor pairs with |r|≥0.75 were considered highly correlated for variable selection.

**Figure 2:**
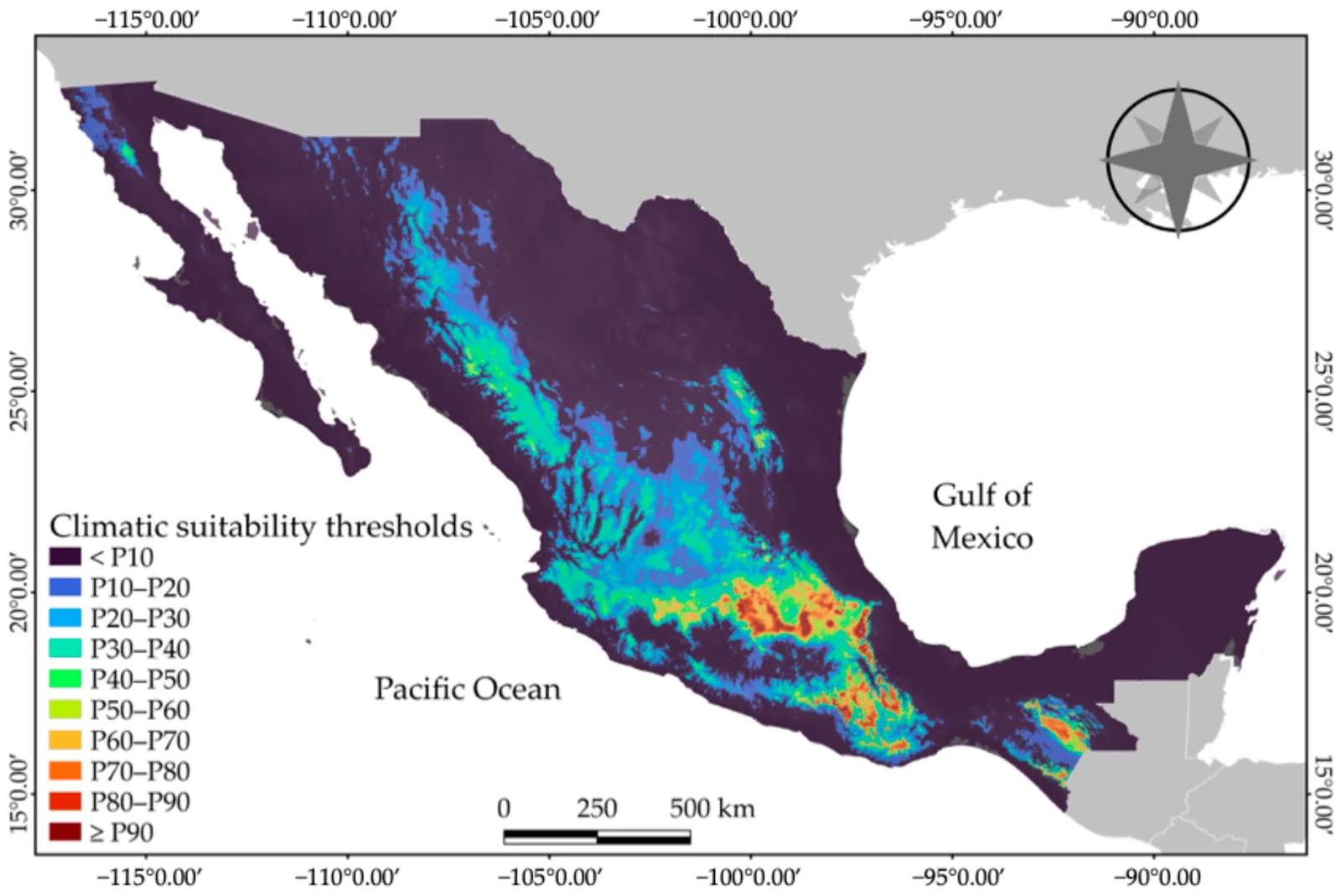
Current climatic suitability of *Castilleja tenuiflora* in Mexico modelled using MaxEnt. Relative climatic suitability is classified using percentile-based thresholds derived from the suitability values of the training presences (<P10, P10–P20, P20–P30, P30–P40, P40–P50, P50–P60, P60–P70, P70–P80, P80–P90, and ≥P90). P10 corresponds to the 10th-percentile training-presence threshold (0.162865), and progressively higher classes indicate increasing climatic suitability. Grey areas represent the cartographic background outside the analyzed raster extent.

**Figure 3:**
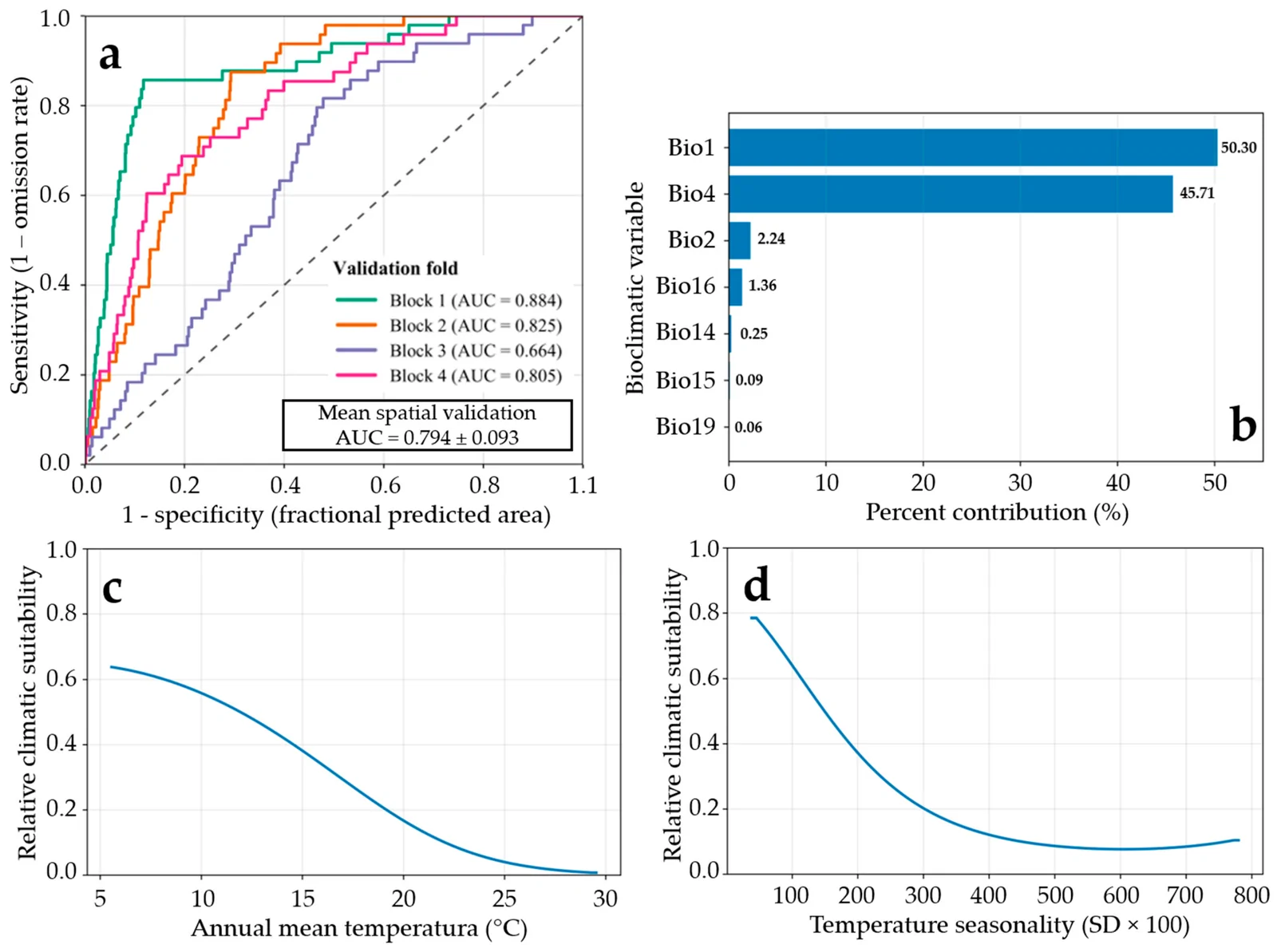
Performance and climatic-variable contributions of the final MaxEnt model for *Castilleja tenuiflora*. (**a**) Receiver operating characteristic (ROC) curves for the four spatial cross-validation folds of the selected model (LQ feature classes; regularization multiplier = 0.5), with a mean spatial-validation AUC of 0.794 ± 0.093. The dashed diagonal line represents the performance expected under random discrimination (AUC = 0.5). (**b**) Percent contribution of the seven bioclimatic variables included in the final model. Bio1: Annual mean temperature (°C); Bio2: Diurnal temperature variation (°C); Bio4: Temperature seasonality (SD × 100); Bio14: Precipitation in the driest period (mm); Bio15: Precipitation seasonality (CV); Bio16: Precipitation in the wettest quarter (mm); Bio19: Precipitation in the coldest quarter (mm). (**c**) Response curve of relative climatic suitability to annual mean temperature (Bio1). (**d**) Response curve of relative climatic suitability to temperature seasonality (Bio4). Response curves were derived from the final model fitted using 194 spatially thinned occurrence records and 10,000 background points within the calibration area.

**Figure 4:**
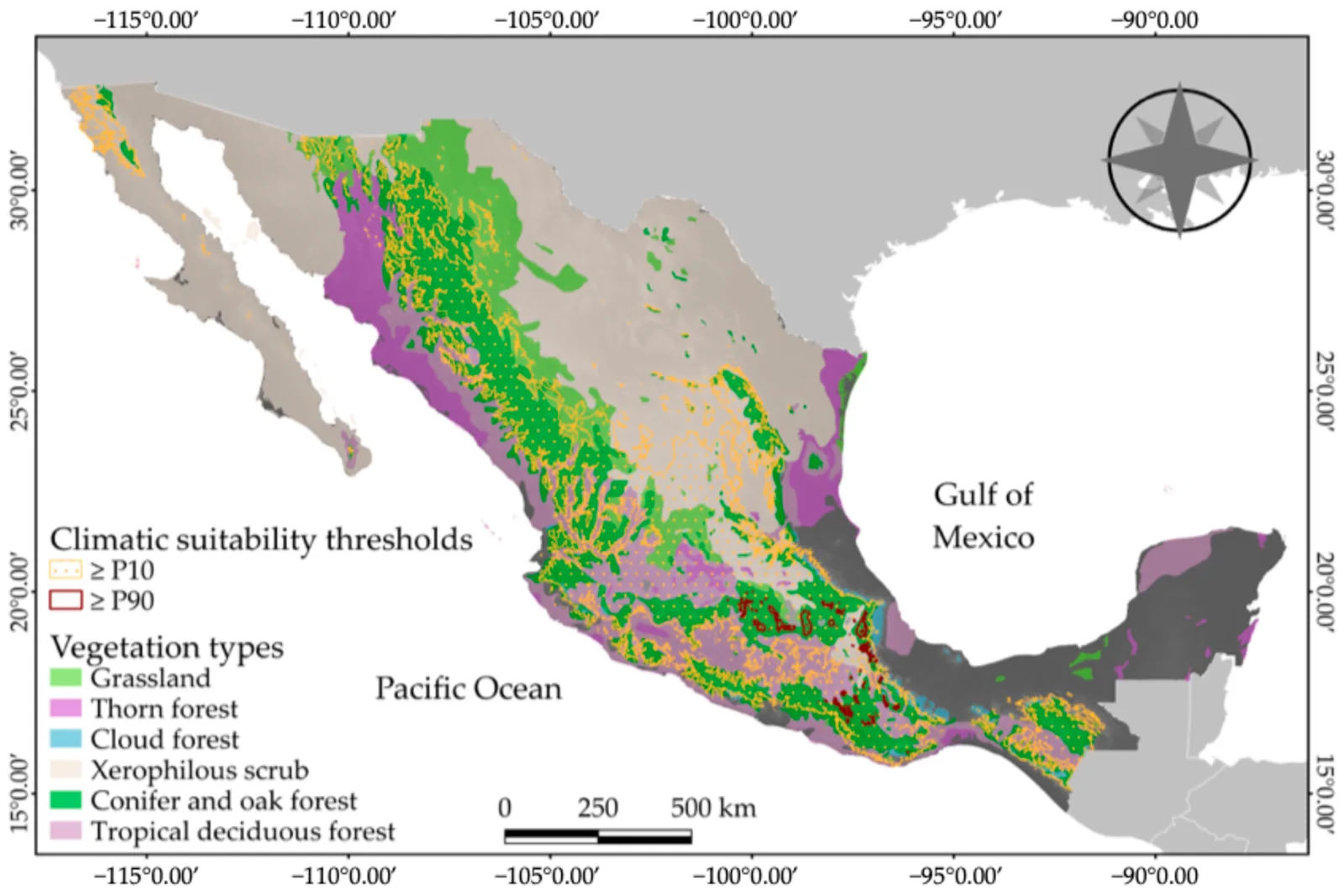
Overlay of vegetation types in Mexico with the current climatic suitability of *Castilleja tenuiflora*. Climatic suitability is shown using the ≥P10 and ≥P90 thresholds for visual clarity, whereas quantitative analyses used the complete P10–P90 classification. The map shows the spatial correspondence between climatically suitable areas and the main vegetation types occurring in Mexico. The vegetation layer is presented as environmental context rather than as an independent validation of the climatic suitability model.

**Figure 5:**
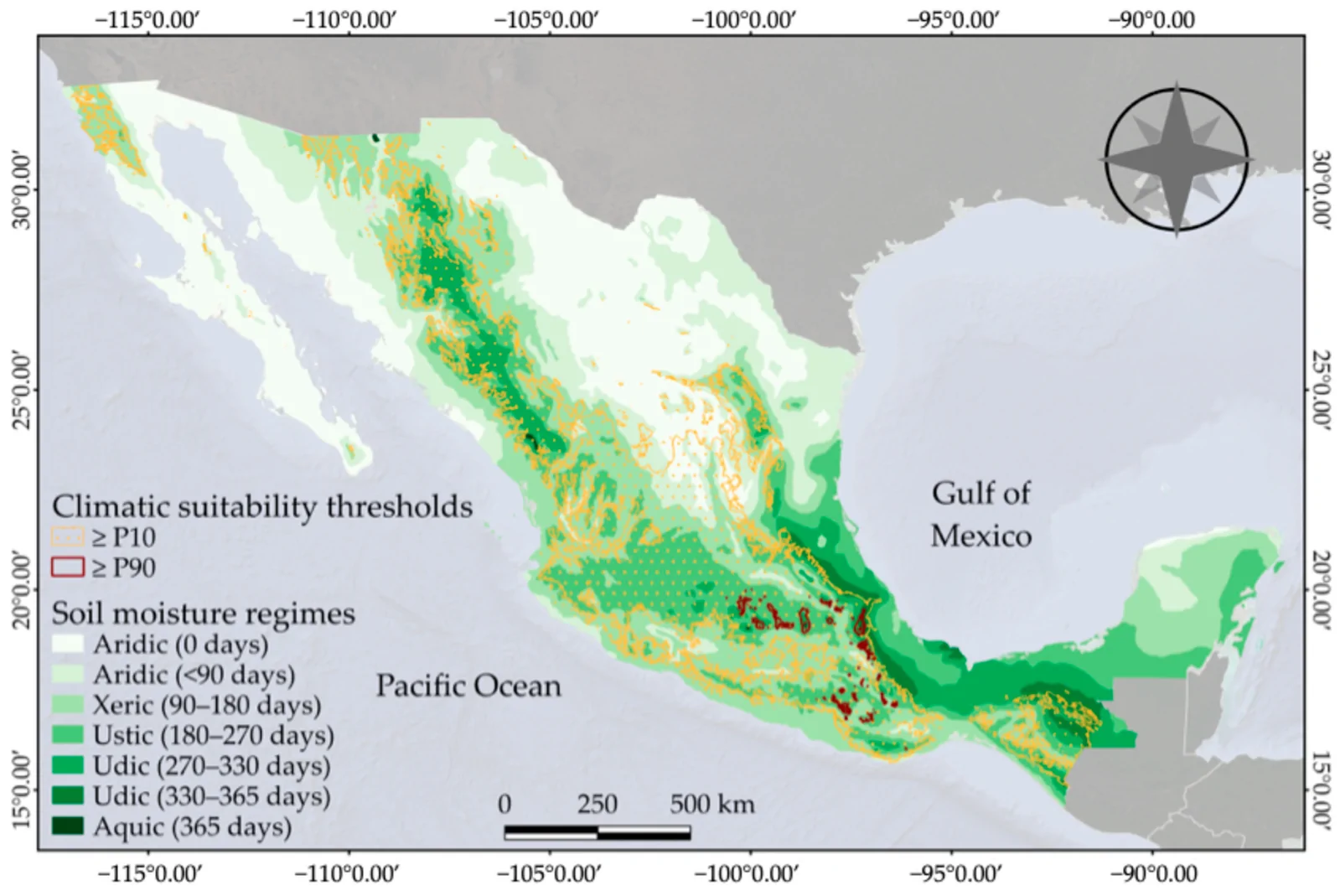
Overlay of soil moisture regimes in Mexico with the current climatic suitability of *Castilleja tenuiflora*. Climatic suitability is shown using the ≥P10 and ≥P90 thresholds for visual clarity, whereas quantitative analyses used the complete P10–P90 classification. Soil moisture regimes range from aridic to aquic conditions according to the approximate number of days per year with available soil moisture. The overlay is presented as environmental context rather than as an independent validation of the climatic suitability model.

**Figure 6:**
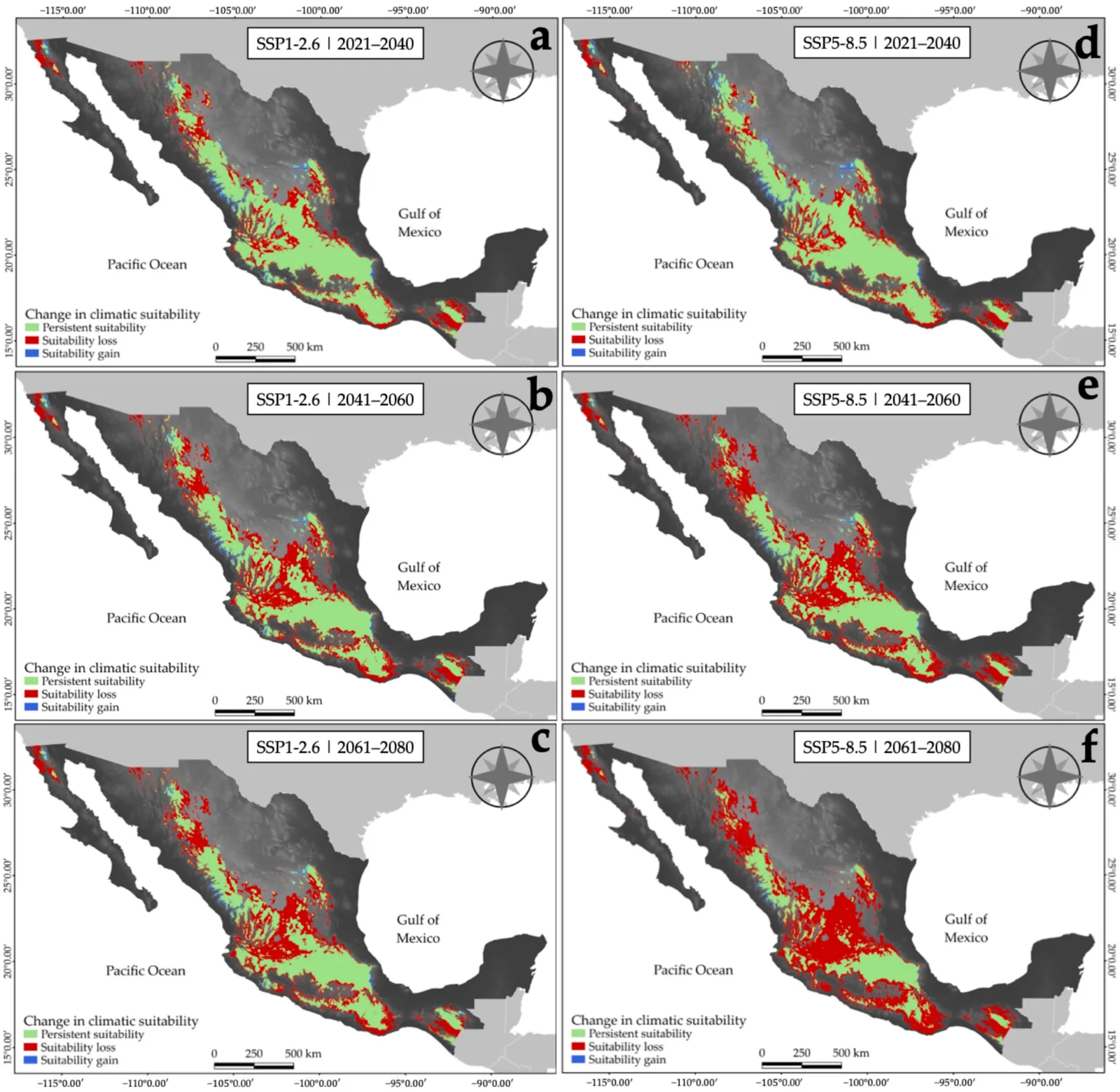
Projected changes in climatic suitability of *Castilleja tenuiflora* in Mexico under SSP1-2.6 and SSP5-8.5. Changes are shown relative to current climatic suitability using the 10th-percentile training-presence threshold (P10 = 0.162865). (**a**–**c**) SSP1-2.6 projections for 2021–2040, 2041–2060, and 2061–2080, respectively; (**d**–**f**) SSP5-8.5 projections for the same periods. Persistent suitability represents areas ≥P10 under both current and future conditions; suitability loss represents areas currently ≥P10 that fall below P10 under future conditions; and suitability gain represents areas currently below P10 that become ≥P10 under future conditions. Areas unsuitable under both current and future conditions are shown as the cartographic background.

**Table 1:** Climate types with the greatest spatial overlap with the current climatic suitability of *Castilleja tenuiflora* in Mexico.

Climate Type	Short Climatic Description	≥P10 Area (km^2^)	≥P90 Area (km^2^)
BS1kw	Temperate semi-arid, with summer rainfall	93,162.67	—
C(w1)	Temperate subhumid, with summer rainfall	53,101.11	534.78
(A)C(wo)	Semi-warm subhumid, with summer rainfall	49,092.23	—
C(w2)	Temperate subhumid, with summer rainfall	48,191.07	853.61
(A)C(w1)	Semi-warm subhumid, with summer rainfall	43,488.74	—
Cb’(w2)	Semi-cold subhumid with a long cool summer	—	3575.31
C(m)	Temperate humid, with summer rainfall	—	104.06
C(m)(f)	Temperate humid, with greater winter rainfall	—	91.43

Only the five climate types with the greatest spatial overlap at the ≥P10 and ≥P90 climatic suitability thresholds are shown. Dashes indicate that a climate type was not among the five highest-ranked categories for that threshold, rather than absence of overlap. Climate types follow the Köppen–García classification. Detailed temperature and precipitation criteria for each climate type are provided in Table S6.

**Table 2:** Projected changes in climatic suitability of *Castilleja tenuiflora* in Mexico under SSP1-2.6 and SSP5-8.5.

SSP	Period	Future ≥P10 (km^2^)	Persistence (km^2^)	Loss (km^2^)	Gain (km^2^)	Net Change (km^2^)	Net Change (%)
SSP1-2.6	2021–2040	410,665.71	397,024.74	151,792.68	13,640.97	−138,151.71	−25.17
SSP1-2.6	2041–2060	335,352.41	326,175.19	222,642.23	9177.22	−213,465.01	−38.90
SSP1-2.6	2061–2080	320,593.60	312,764.56	236,052.86	7829.04	−228,223.82	−41.58
SSP5-8.5	2021–2040	430,744.06	410,356.09	138,461.33	20,387.96	−118,073.36	−21.51
SSP5-8.5	2041–2060	289,165.89	284,192.39	264,625.03	4973.51	−259,651.53	−47.31
SSP5-8.5	2061–2080	190,692.25	188,745.81	360,071.61	1946.44	−358,125.17	−65.25

Future climatically suitable area was quantified using the 10th-percentile training-presence threshold (P10 = 0.162865) derived from the current MaxEnt model and applied consistently to all future projections. Persistence represents areas ≥P10 under both current and future conditions; loss represents currently suitable areas that fall below P10 in the future; and gain represents areas currently below P10 that become ≥P10 under future conditions. Net change was calculated as gain minus loss, and net change (%) expresses the difference between future and current ≥P10 area relative to the current reference area (548,817.42 km^2^). All present–future comparisons were calculated within the common valid raster domain. Future projections were generated using CMCC-ESM2 for SSP1-2.6 and SSP5-8.5.

**Table 3:** Elevational distribution of current and projected climatic suitability for *Castilleja tenuiflora* under SSP1-2.6 and SSP5-8.5.

Scenario	Period	≥P10	≥P50	≥P70	≥P90
Current	1970–2000	2011	2443	2654	3102
SSP1-2.6	2021–2040	2114 (+103)	2626 (+184)	2879 (+225)	3552 (+450)
SSP1-2.6	2041–2060	2176 (+164)	2669 (+226)	2953 (+299)	3585 (+483)
SSP1-2.6	2061–2080	2185 (+173)	2637 (+195)	2881 (+228)	3444 (+342)
SSP5-8.5	2021–2040	2115 (+104)	2619 (+177)	2854 (+200)	3483 (+381)
SSP5-8.5	2041–2060	2222 (+210)	2732 (+289)	3047 (+393)	3713 (+611)
SSP5-8.5	2061–2080	2342 (+331)	2893 (+450)	3346 (+692)	3931 (+829)

Values represent the mean elevation (m a.s.l.) of areas meeting or exceeding each climatic suitability threshold. Values in parentheses indicate the elevational difference relative to current conditions for the corresponding threshold; positive values indicate an increase in elevation. Current conditions correspond to the 1970–2000 climatic baseline. P10, P50, P70, and P90 correspond to progressively more restrictive percentile-based climatic suitability thresholds derived from training-presence suitability values.

**Table 4:** Current and projected climatic suitability of *Castilleja tenuiflora* within the Mexican Protected Natural Area network under SSP1-2.6 and SSP5-8.5.

Scenario	Period	Threshold	Area in PNAs (km^2^)	National Suitability Represented (%)	Change vs. Current ≥P10 (%)
Current	1970–2000	≥P10	48,689.93	8.87	0.00
		≥P50	6497.76	8.51	—
		≥P70	3786.53	12.71	—
		≥P90	1545.69	28.69	—
SSP1-2.6	2021–2040	≥P10	38,486.26	9.37	−20.96
		≥P50	5092.19	14.96	—
		≥P70	2752.28	28.35	—
		≥P90	379.50	54.35	—
SSP1-2.6	2041–2060	≥P10	34,886.86	10.40	−28.35
		≥P50	4531.99	16.71	—
		≥P70	2487.37	31.63	—
		≥P90	456.06	58.48	—
SSP1-2.6	2061–2080	≥P10	34,278.91	10.69	−29.60
		≥P50	4624.02	15.10	—
		≥P70	2924.56	26.80	—
		≥P90	763.72	50.42	—
SSP5-8.5	2021–2040	≥P10	40,832.18	9.48	−16.14
		≥P50	5050.59	14.42	—
		≥P70	2595.53	25.38	—
		≥P90	359.94	50.44	—
SSP5-8.5	2041–2060	≥P10	30,970.42	10.71	−36.39
		≥P50	3994.50	19.64	—
		≥P70	1827.32	34.95	—
		≥P90	341.82	65.60	—
SSP5-8.5	2061–2080	≥P10	23,638.25	12.40	−51.45
		≥P50	2799.09	25.89	—
		≥P70	850.24	42.97	—
		≥P90	251.75	74.97	—

Areas within Protected Natural Areas (PNAs) are reported for the ≥P10, ≥P50, ≥P70, and ≥P90 climatic suitability thresholds. P10 corresponds to the 10th-percentile training-presence threshold (0.162865), whereas P50, P70, and P90 represent progressively more restrictive percentile-based thresholds derived from suitability values at the training presences. “National suitability represented (%)” indicates the proportion of the total climatically suitable area in Mexico at each threshold that overlaps the PNA network. “Change vs. current ≥P10 (%)” expresses the percentage change in PNA area above P10 relative to the current reference value of 48,689.93 km^2^; this comparison is reported only for the ≥P10 threshold. Current conditions correspond to the 1970–2000 climatic baseline, whereas future projections were generated using CMCC-ESM2 under SSP1-2.6 and SSP5-8.5 for 2021–2040, 2041–2060, and 2061–2080.

## Data Availability

The data supporting the findings of this study are available from the corresponding author upon reasonable request.

## Supplementary Materials

The following supporting information can be downloaded at: https://www.mdpi.com/article/10.3390/plants15192935/s1, Figure S1. Geographical distribution of the 278 cleaned occurrence records of *C. tenuiflora* used to define the accessible area (M) in Mexico. Records were retained after data cleaning and removal of duplicate presences occurring within the same environmental raster cells, prior to the 5 km and 10 km spatial-thinning procedures used for model calibration and sensitivity analysis, respectively; Table S1. Provenance and retention of occurrence records of *Castilleja tenuiflora* after hierarchical spatial thinning; Table S2. Bioclimatic predictor screening and final selection for the climatic-suitability model of *Castilleja tenuiflora*; Table S3. Complete ENMeval tuning results for 40 candidates MaxEnt parameterisations evaluated independently after 5 km and 10 km spatial filtering of *Castilleja tenuiflora* occurrence records; Table S4. Comparison of the selected MaxEnt models obtained after 5 km and 10 km spatial filtering; Table S5. Fold-specific training and validation AUC values from spatial block cross-validation of the selected MaxEnt models of *Castilleja tenuiflora*; Table S6. Spatial overlap between current climatic suitability of *Castilleja tenuiflora* and Mexican climate categories; Table S7. Sensitivity of national climatic-suitability area to progressively more restrictive percentile-based thresholds; Table S8. Federal Protected Natural Areas recurrently containing climatically suitable area under current conditions and all six future projections; Table S9. Representation of current and future climatic suitability within the Mexican Protected Natural Area network.

## Abbreviations

The following abbreviations are used in this manuscript:

CMIP6	Coupled Model Intercomparison Project Phase 6
SSP1-2.6	Shared Socioeconomic Pathway 1-2.6
SSP5-8.5	Shared Socioeconomic Pathway 5-8.5
PNAs	Protected Natural Areas
MPNAs	Mexican Protected Natural Areas
