# Supplementary material for: Present and Future Climatic Suitability of Castilleja tenuiflora in Mexico
Source: Plants (Basel). 2026 Sep 25;15(19):2935. doi: 10.3390/plants15192935 (PMC13667020; doi:10.3390/plants15192935)
Supplement: Supplementary file 1 [file plants-15-02935-s001.zip › Figure S1.pdf]

Supplementary Materials

Luis Rafael Garibay-Castro, Alma Rosa López-Laredo, Elizabeth Rubio-Rodríguez,Gabriela Trejo-Tapia and José Luis Trejo-Espino

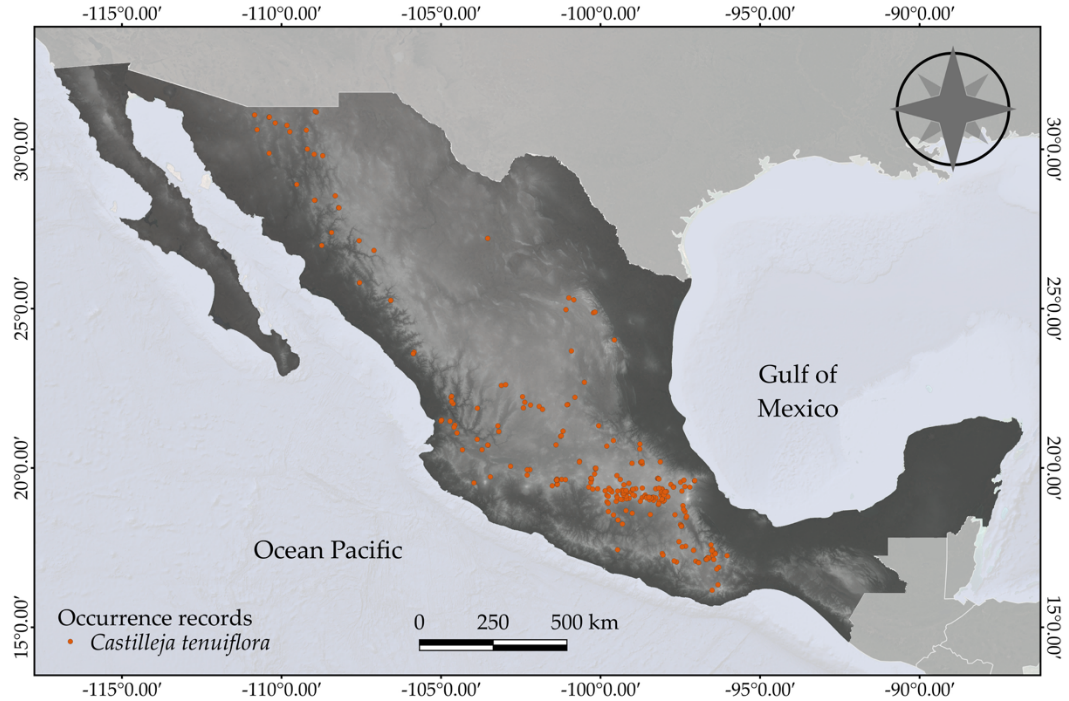

Figure S1. Geographical distribution of the 278 cleaned occurrence records of *C. tenuiflora* used to define the accessible area (M) in Mexico. Records were retained after data cleaning and removal of duplicate presences occurring within the same environmental raster cells, prior to the 5-km and 10-km spatial-thinning procedures used for model calibration and sensitivity analysis, respectively.
